# Psychometric evaluation of the Chinese version of the fear of intimacy with helping professionals scale

**DOI:** 10.1371/journal.pone.0196774

**Published:** 2018-05-24

**Authors:** Ying Lau, Kin Sun Chan

**Affiliations:** 1 Department of Alice Lee Centre for Nursing Studies, Yong Loo Lin School of Medicine, National University of Singapore, Singapore; 2 Faculty of Social Sciences and Humanities, the University of Macau, Macau Special Administration Region, China; Hong Kong Polytechnic University, HONG KONG

## Abstract

**Objectives:**

This study aimed to evaluate the internal consistency, reliability, convergent validity, known-group comparisons, and structural validity of the Chinese version of Fear of Intimacy with Helping Professionals (C–FIS–HP) scale in Macau.

**Methods:**

A cross-sectional design was used on a sample of 593 older people in 6 health centers. We used Chinese version of Exercise of Self-Care Agency Scale (C-ESCAS) and Morisky 4-item medication adherence scale to evaluate self-care actions and medication adherence. The internal consistency and reliability of C–FIS–HP were analyzed using the Spearman-Brown split-half reliability, Cronbach’s alpha, and test–retest reliability. Convergent validity was tested the construct of C–FIS–HP and self-care actions. Known-group comparisons differentiated predefined groups in an expected direction. Two separated samples were used to test the structural validity. An exploratory factor analysis (EFA) tested the factor structure of C–FISHP using the principal axis factoring. A confirmatory factor analysis (CFA) was further conducted to confirm the factor structure constructed in the prior EFA.

**Results:**

The C–FIS–HP had a Spearman-Brown split-half coefficient, Cronbach’s alpha, and intraclass correlation coefficient of 0.96, 0.93, and 0.96, respectively. Convergent validity was satisfactory with significantly correlations between the C-FIS-HP and C-ESCAS. C–FIS–HP to differentiate the differences between high-, moderate-, and low- medication adherence groups. EFA demonstrated a two-factor structure among 297 older people. A first-order CFA was performed to confirm the construct dimensionality of C–FIS–HP with satisfactory fit indices (NFI = 0.92; IFI = 0.95; TLI = 0.94; CFI = 0.95 and RMSEA = 0.07) among 296 older people.

**Conclusions:**

C–FIS–HP is a reliable and valid test for assessing helping relationships in older Chinese people. Health professionals can use C–FIS–HP as a clinical tool to assess the comfort level of patients in a helping relationship, and use this information to develop culturally sensitive therapeutic interventions and treatment plans. Further studies need to be conducted concerning the different psychometric properties, as well as the application of C–FIS–HP in various regions.

## Introduction

The helping relationship is the heart of the helping process, and is essential in delivering health services and treatment [1]. Over the past 50 years, helping relationships have become multidimensional and considerably crucial to health issues [2,3]. The helping process depends on an open, honest, caring, and empathic relationship between patients and their health professionals [1,4]. Positive helping relationships have significant effect on health outcomes [5], recovery process [6], perceived self-care agency [7,8], and medication adherence [9]. Thus, measuring the helping relationship is important to understand the comfort level of patients in terms of sharing their condition or feelings to health care professionals, who can predict the help-seeking behaviors of the former [10].

Cultural differences influence helping relationships [11,12]. Chinese cultural values emphasize interdependence, humility, emotional restraint, avoidance of shame, concern for face, and preservation of family honor [13,14], thereby possibly inducing discomfort in disclosing personal information to health professionals. The Chinese generally prefer to keep personal matters within the family rather than share such issues with outsiders; thus, this attitude may shape their help-seeking behaviors [15,16]. The ability to understand the helping relationships among the Chinese population may illuminate potential strategies to improve their health outcomes [5,17]. Therefore, a culturally relevant and valid assessment tool is necessary to understand helping relationships in the Chinese population.

Assessment tools that address helping relationships are relatively scarce. A few of these tools are limited to self-developed measures without reporting the psychometric properties [18] or solely used for pharmacists and patient relationships [19] or patients with HIV [20]. The Fear of Intimacy with Helping Professionals Scale (FIS–HP) was developed to assess the comfort level of an individual in disclosing intimate personal details to helping professionals [10]. FIS–HP is a modified version of the original 35-item Fear of Intimacy Scale (FIS) [21]. Intimacy refers to close interpersonal relationship [21], whereas fear of intimacy refers to the inhibited capability of an individual to interchange significant personal thoughts and feelings with other individuals [21]. The items in the original FIS was based on the assumption that feat of intimacy interfered the ability to interchange feelings and thoughts [21]. The 18 items of FIS-HP were developed from the original 35 items for latter’s applicability to the professional helping relationship [10].

The Chinese version of 18-item FIS (i.e., C–FIS–HP) was developed in a sample of 150 older people living in Mainland China to assess the comfort level of an individual in disclosing intimate personal details to a helping professional [10]. Individuals have limited satisfaction with their current relationships if they felt uncomfortable toward helping professionals are [10]. Consequently, the prospects of developing long-term relationships become less plausible [10]. Undoubtedly, understanding the comfort level of a client about sharing information with a helping professional is considerably beneficial in the helping process [1,10]. Both FIS-HP and C-FIS-HP reported a three-factor structure among mainland Chinese and American older people, namely, “fear of sharing,” “openness to intimate sharing,” and “information sharing” [10].

Although C–FIS–HP [10] was developed within the Mainland China population, a few significant limitations have been determined. First, a small sample size (N = 150) was used in previous study [10] and a sample size of at least 200 may be necessary for exploratory factor analysis (EFA) [22]. Second, principle component analysis (PCA) was used as a factor-extraction procedure in a previous study [10]; however, the use of PCA is still a debatable method of determining underlying dimensions [22]. PCA does not differentiate between common and unique variance, and tends to produce inaccurate results and generate misleading factor loading [22]. Third, evidence is lacking for construct equivalence through confirmatory factor analyses (CFA). Accordingly, EFA has been used to explore the possible underlying factor structure without imposing a preconceived structure [22]. By contrast, CFA, which is a superior statistical technique, has been used to confirm the factor structure [23]. To the best of our knowledge, no further psychometric testing on C–FIS–HP has been conducted up to the present. Successive verifications in different population were necessary to confirm the reliability and validity of C–FIS–HP [24]. Therefore, the purpose of the present study was to evaluate the psychometric properties of C–FIS–HP among older Chinese people in Macau, China.

## Methods

### Design

A cross-sectional design was used in 593 older people. Test–retest reliability, item–total correlation test, Cronbach’s alpha, and Spearman-Brown split half reliability were used to test the internal consistency and stability of C–FIS–HP. Convergent, known-group comparisons, EFA, and CFA were used to test the construct validity of C–FIS–HP.

### Setting and sample

The research setting was in Macau Chinese Special Administrative Region, which is located on the southeastern coast of China and with a total land area of 30.4 km^2^ [25]. The total population of Macau in 2015 was 646,800; 95% of the current population are Chinese, 2% are Portuguese, and 3% other groups. Geographically distributed cluster samples from six health centers in Macau, were used. Six health centers provide community health services in Macau including adult health care, oral health care, family care, prenatal care, student health, health education and Traditional Chinese Medicine service. Our target population focused on older people (i.e., aged 55 years and older) [26] because the onset of common chronic diseases were reported among this age group [27]. Older people served by the six health centers under the Health Bureau of Macau were identified as representative of the general population in Macau. A minimum desirable sample size of 200 for EFA was recommended to obtain factor solutions that are adequately stable and near the population factors [22,28]. Thus, we used a sample size of 297 participants for EFA in the current study. A minimum necessary sample size for CFA was 180 based on the proposed ratios of sample size to parameter estimates of 10 to 1 [29]; therefore, we used a sample size of 296 participants. The inclusion criteria for the samples were as follows: (1) older people (age ≥ 55 years old), (2) able to communicate in Chinese, and (3) can supply written informed consent. The exclusion criteria were as follows: (1) severe aphasia or dysphasia and (2) severe impaired hearing or vision that may affect the quality of responses.

### Data collection

Permission was obtained from the original authors of C–FIS-HP. This study was reviewed and approved by Macau Health Bureau Ethics Committee. The participants had scheduled medical appointments in the six health centers. One experienced research assistant was screened for eligibility and invited to participate in this study. The informed consents were obtained after providing an explanation of the current study. Thumbprint was accepted as signature for illiterate older people. To collect the study data, the research assistant conducted face-to-face interviews inside a single room.

### Measures

Demographic variables (e.g., age, gender, education, and marital status), multiple chronic conditions (i.e., number of chronic diseases), and three validated measures were collected.

#### Fear of intimacy with helping professionals scale

The 18-item FIS-HP [10] was used to assess the comfort level of older adults in disclosing intimate details with health care professionals. The items use a 5-point Likert scale ranging from 1 (not at all characteristic of me) to 5 (extremely characteristic of me); 18–90 was the range of the total score. Individuals with high FIS–HP scores indicated feeling less comfortable toward helping professionals [10]. C–FIS–HP has satisfactory convergent validity [10] and reported a three-factor structure, namely, “fear of sharing,” “openness to intimate sharing,” and “information sharing” [10]. Cronbach’s alpha was 0.88, thereby indicating satisfactory internal consistency [10].

#### Exercise of self-care agency scale

The 43-item Exercise of Self-Care Agency Scale (ESCAS) was used to measure a person’s agency or power to engage in self-care actions [30]. Each item is scored from 0 (very uncharacteristic of me) to 4 (very characteristic of me). The maximum score of the instrument is 172, which indicates a high degree of exercise of self-care agency. The original English version has shown a test–retest reliability of 0.77 and split–half reliabilities of 0.80 and 0.81 [30]. Alpha reliability coefficients ranged from 0.77 to 0.92, test–retest reliability coefficient ranged from 0.81 to 0.91, and construct validity of ESCAS (C-ESCAS) in the Chinese version were satisfactory [31,32].

#### Morisky 4-item medication adherence scale

The Morisky 4-item medication adherence scale (MMAS–4) was used to assess the extent of the adherence of patients to medical regimens [33]. The items are rated on a dichotomous response format (i.e., yes/no) and the sum of the “yes” answers provided a composite measure of non-adherence. The total score ranged from 1 to 4, with a considerably high score indicating an improved medication adherence. The participants who had scores of 4, 2 to 3, and 0 to 1 were classified as the high-, moderate, and low-adherence groups, respectively [34]. The Chinese version of the MMAS–4 (C–MMAS–4) demonstrated satisfactory known-group validity, construct and criterion validities, and good internal consistency (α = 0.73) [34].

### Data analysis

IBM SPSS 24.0 (IBM Corporation, Armonk, NY, USA) was used to analyze the data. Descriptive statistics was used to analyze the demographic variables, multiple chronic conditions, and scores of C–FIS–HP, C–ESCAS and C–MMAS–4.

#### Reliability

The test–retest reliability, item–total correlation test, Cronbach’s alpha, and Spearman-Brown split half reliability were used to test the reliability of C–FIS–HP. The test–retest reliability was used to test the stability of the scale between 50 subsample older people who had stable helping relationship (> 3 months) using the intraclass correlation coefficient (ICC) at an interval of three weeks during their follow-up visit. We selected 50 older people from one of six health centers. ICC was calculated in two sets of the C–FIS–HP scores, with correlation coefficients ≥ 0.7 [35] and item-to-total correlation coefficients > 0.3 [2] taken as the criterion value. Cronbach’s alpha represents reliability to measure internal consistency, which refers to result consistency delivered in a test [36]. Odd-even splits were used to determine the split-half reliabilities [36, 37]. Split-half method purports to measure the equal contribution of the construct into two sets and the Spearman-Brown formula provides an estimate, based on the split-half correlation, of the reliability of the test as a whole [36]. The Spearman-Brown formula was used to adjust the correlation coefficient for preventing a decrease in the observed reliability of C-FIS-HP when items is split into two parts [36, 37]. The Cronbach’s alpha [36] and split–half methods [37] were used to measure the internal consistency of C–FIS–HP; the coefficient ≥ 0.70 was considered satisfactory [35].

#### Validity

Three different types of construct validity were used: convergent, known-group, and structural validities. Convergent validity measures construct in one measure that theoretically should be related to another measure [24, 36]. In this study, we tested convergent validity by investigating the construct of self-care agency which should theoretically relate to the construct of helping relationship [7, 8]. The Pearson product–moment correlation coefficient was performed for the C–FIS–HP and C–ESCAS. We expected a correlation coefficient in the range of 0.2 to 0.4 for convergent validity [38]. Known-group validity hypothesizes that certain groups of respondents will score higher on a scale than others with known attributes [39]. Moreover, known-group validity can discriminate between individuals based on known differences in the helping relationship of medication adherence [9] and multiple chronic conditions [40,41].

Three groups were predefined based on the C–MMAS–4 scores: high-adherence group (score of 4), moderate-adherence group (score of 2 to 3), and low-adherence group (score of 0 to 1) [34]. Known-group comparisons can obtain significantly different test scores in the expected direction [24]. C–FIS–HP can differentiate the differences between high-, moderate-, and low-adherence groups. The C–FIS–HP score was expected to be the lowest in the high-adherence [9], moderate-adherence [42], and low-adherence groups [43]. The analysis of variance (ANOVA) test was used to compare the group differences between the three groups. By contrast, the known-group comparison analysis was used to discriminate multiple chronic conditions in the subgroups of older people. The C–FIS–HP score was expected to be considerably low in older people with multiple chronic conditions (number of chronic illnesses ≥ 5) [40,41]. Two groups of older people (≥ 5 vs. < 5 chronic illnesses) were compared using the independent *t* test.

To test the structural validity using EFA and CFA, two separated samples were used. The entire sample (N = 593) was randomly divided into two separated files (n = 297 and n = 296). Considering too few or too many factors can have dire consequences on the interpretation of factor pattern, we used a parallel analysis (Eigenvalue Monte Carlo Simulation) [44], a scree plot [45] and empirical findings [10] to decide on the optimal numbers of factors. Parallel analysis involved comparing the actual eigenvalues with the random data eigenvalues using principal components analysis procedure [44]. A series of EFAs was conducted to test the factor structure of C–FIS–HP using principal axis factoring (PAF) with a varimax rotation between 297 older people. In PAF, the analysis of data structure focused on shared variances, which are unique to individual measurements [22,46]. The rotation method encompassed the varimax rotation that rotated factors in multidimensional possibilities to reach the best simple structure [22]. A factor loading (λ) > 0.3 was considered for each variable onto each factor [47]. Bartlett’s test of sphericity verified an identity correlation matrix for the factor analysis, and *P* < 0.05 indicated suitability for structure detection [48]. The proportion of variance in our variables was tested using the Kaiser–Meyer–Olkin (KMO) measure of sampling adequacy. A KMO with 0.6 was recommended as the minimum value for good factor analysis [49].

CFA was performed to validate the factor structure constructed in the prior EFA between another 296 older people [50]. In this study, we planned to compare the fit indices between our model and three-factor model as suggested in the previous study [10]. In order to compare underlying construct, a bi-factor model was used to understand whether unique variance over and above the common variance in helping relationship reporting explained by a general factor [20]. A potential function of a bi-factor model is to provide a conceptual clarity by a separation between general and specific variance [51]. The Analysis of Moment Structures (AMOS) 24.0 software was used to test 2-factor, 3-factor and bi-factor models by CFA. The following model goodness-of-fit indices were used to evaluate the model fit: normed fit index (NFI), incremental fit index (IFI), Tucker–Lewis index (TLI), comparative fit index (CFI), and root means square error of approximation (RMSEA) [48,52]. The following cut-off criteria for the fit index were used: (1) NFI > 0.90; (2) IFI > 0.90; (3) TLI > 0.90; (4) CFI > 0.90, and (5) RMSEA < 0.08 [53, 54].

## Results

A total of 734 eligible older people were recruited from six health centers; 593 older people eventually accepted our invitation (response rate: 80.8%). Table 1 shows the descriptive analyses of the demographic variables, duration of helping relationship, multiple chronic conditions, and scores of C–FIS–HP, C–ESCAS, and C–MMAS–4. The mean age of the participants was 66 (SD = 8.03) and ranged from 55 to 91 years. Approximately half of the participants were male (50.3%) and relatively the same percentage had primary or lower education level (56%). Most of them (84.3%) had a stable helping relationship with health-care profession (> 3 months). The majority of the participants were married (78.9%) and had over one chronic disease (62.1%).

**Table 1:** Descriptive analyses of participant’s characteristics, multi-morbidities, medication adherence, relationships with helping professionals, and self-care ability (N = 593).

Participants characteristics			
Age [M(SD)]			66 (8.03)
Gender [n (%)]			
		Female	295 (49.7)
		Male	298 (50.3)
Education [n (%)]			
		≥ Secondary	261 (44.0)
		≤ Primary	332 (56.0)
Marital Status [n (%)]			
		Married	468 (78.9)
		Others	125 (21.1)
Duration of helping relationship			
		1–3 months	93 (15.7)
		> 3 months	500 (84.3)
Multi-morbidities			
	Numbers of chronic illnesses [n (%)]		
		≤1	225 (37.9)
		2	168 (28.3)
		3	92 (15.5)
		4	54 (9.1)
		≥5	54 (9.1)
Medication adherence			
	C-MMAS-4 [n (%)]		
		High adherence	105 (17.7)
		Moderate adherence	301 (50.8)
		Low adherence	187 (31.5)
Relationships with helping professionals			
	C-FIS-HP [M(SD)]		24.46 (15.01)
Self-care ability			
	C-ESCAS [M(SD)]		133.33 (19.58)

M(SD) = mean (standard deviation); n (%) = number (percentage); C-FIS-HP = Chinese version of the Fear of Intimacy with Helping Professionals scale; C-MMAS-4 = Chinese version of the Morisky 4-Item Medication Adherence Scale; C-ESCAS = Chinese version of the Exercise of Self-Care Agency scale.

### Reliability

Table 2 shows the internal consistency and test–retest reliability of C-FIS-HP. The item-to-total correlation of the two subscales ranged from 0.37 to 0.87, thereby suggesting an acceptable internal consistency. Spearman-Brown split half reliability and Cronbach’s alpha for subscales and total scale of C–FIS–HP were 0.75 to 0.97, respectively, thereby indicating satisfactory reliability. ICCs of the total and subscales were above 0.95, which suggested good test–retest reliability.

**Table 2:** Internal consistency (item-to-total correlations, Spearman-Brown coefficient, and Cronbach’s alpha coefficients) and test-retest reliability of Chinese version of the Fear of Intimacy with helping professionals scale.

Items		Item-to-total correlation	Spearman-Brown coefficient (N = 593)	Cronbach’s alpha (N = 593)	Intraclass coefficient (n = 50)
	**Subscale: Willingness of sharing**		0.97^a^	0.96	0.97
FIS12	I would be comfortable revealing to HP what I feel are my shortcomings and handicaps.	0.87			
FIS11	I would feel comfortable trusting HP with my deepest thoughts and feelings.	0.86			
FIS16	I would feel comfortable about having open and honest communication with HP.	0.86			
FIS15	I would be comfortable telling HP what my needs are.	0.85			
FIS10	I would feel comfortable telling HP things that I do not tell other people.	0.84			
FIS9	I would feel comfortable not sharing very personal information with HP.	0.83			
FIS5	I would be comfortable discussing significant problems with HP.	0.80			
FIS8	I would not be afraid to share with HP what I dislike about myself.	0.79			
FIS6	I would feel comfortable telling my experiences, even sad ones, to HP.	0.79			
FIS18	I would feel comfortable talking about my personal goals with HP.	0.78			
FIS3	I would feel comfortable expressing my true feelings to HP.	0.75			
FIS17	I would feel at ease to completely be myself around HP.	0.54			
	**Subscale: Fear of sharing**		0.75^b^	0.75	0.95
FIS13	I would be afraid of sharing my private thoughts with HP.	0.57			
FIS4	I might be afraid to confide my innermost feelings to HP.	0.59			
FIS14	I would be afraid that I might not always feel close to HP.	0.56			
FIS7	I would find it difficult being open with HP about my personal thoughts.	0.50			
FIS2	I would feel uneasy talking with HP about something that has hurt me deeply.	0.35			
FIS1	I would feel uncomfortable telling HP about things in the past that I have felt ashamed of.	0.37			
	**Total scale: Chinese version of the Fear of Intimacy with Helping Professionals scale**		0.96^c^	0.93	0.96

^a^ Split half for willingness of sharing subscale: First half items including FIS 3, 5, 9, 11, 15, 17 and second half items including FIS 6, 8, 10, 12, 16, 18

^b^ Split half for fear of sharing subscale: First half items including FIS 1, 7, 3 and second half items including FIS 2, 4, 14

^c^ Split half for overall scale: First half items including FIS1, 3, 5, 7, 9, 11, 13, 15, 17 and second half items including FIS 2, 4, 6, 8, 10, 12, 14, 16, 18

### Convergent validity

The correlation between C–FIS–HP and C–ESCAS was determined by convergent validity. C–FIS–HP was assumed to correlate negatively with this measure [7,8]. Pearson product-moment correlation coefficient between the C-FIS-HP and C-ESCAS showed significantly negatively correlated (−0.422; *P* < 0.0001), thereby showing satisfactory convergent validity [38].

### Known-group comparisons

Levels of medication adherence [9] and multiple chronic conditions [40,41] in the older people subgroups were discriminated using the known-group comparison analysis. Table 3 shows the comparisons of mean scores of C-FIS-HP in different groups. One-way ANOVA with post-hoc Tukey Honestly Significant Difference (HSD) test was used to compare the proportions of three medication adherence groups (20.03, 22.33, and 27.87 in the high, moderate, and low medication adherence groups, respectively), which showed statistically significant differences (*P* <0.0001). Independent sample *t* test was used to compare the proportions of two groups of chronic conditions (24.25 vs. 17.83 between those with ≥ 5 and those with < 5 chronic illnesses, respectively), which were significantly different in terms of statistics (*P* = 0.007). Therefore, C–FIS–HP supports the known-group validity, thereby reflecting the individual differences in the trait being measured [24,39].

**Table 3:** Comparison of the C-FIS-HP scores by C-MMAS-4 and multi-morbidities (N = 593).

		**M(SD)**	***F*- value**	**df**	***P* value**
C-MMAS-4	High adherence	20.03 (16.99)	8.829	2, 590	<0.0001^a^
	Moderate adherence	22.33 (17.53)			
	Low adherence	27.87 (16.77)			
		**M(SD)**	***t*- value**	**df**	***P* value**
Multi-morbidities	Numbers of chronic illness < 5	24.25 (17.46)	2.769	1, 591	0.007^b^
	Numbers of chronic illness ≥5	17.83 (16.12)			

M(SD) = mean (standard deviation)

^a^ One-way ANOVA with post-hoc Tukey HSD test

^b^ Independent sample *t–*test; df = degree of freedom; C-FIS-HP = Chinese version of the Fear of Intimacy with Helping Professionals scale; C-MMAS-4 = Chinese version of the Morisky 4-Item Medication Adherence Scale.

### Exploratory factor analysis

Table 4 summarizes component, raw data eigenvalues, mean and percentile random data eigenvalues of the C-FIS-HP using principal components analysis for normal distributed random data generation parallel analysis [44]. It was clear that the first two eigenvalues from the raw data were larger the corresponding first two 95^th^ percentile (and mean) random data eigenvalues. However, the third eigenvalue from the raw data was less than the third 95^th^ percentile (and mean) random data eigenvalue. The result indicated that two factors should be retained [44].

**Table 4:** Raw data eigenvalues, mean and percentile random data eigenvalues of the C-FIS-HP (n = 297).

Factor	Raw data eigenvalues	Mean	95% of eigenvalues
1	9.01	1.46	1.54
2	2.04	1.36	1.43
3	1.04	1.30	1.35
4	0.82	1.24	1.28
5	0.77	1.19	1.23
6	0.66	1.13	1.18
7	0.61	1.09	1.13
8	0.52	1.05	1.08
9	0.47	1.00	1.04
10	0.37	0.96	1.00
11	0.34	0.92	0.95
12	0.30	0.88	0.92
13	0.26	0.84	0.88
14	0.24	0.8	0.84
15	0.20	0.76	0.8
16	0.16	0.72	0.76
17	0.11	0.67	0.71
18	0.07	0.62	0.67

**Fig 1:**
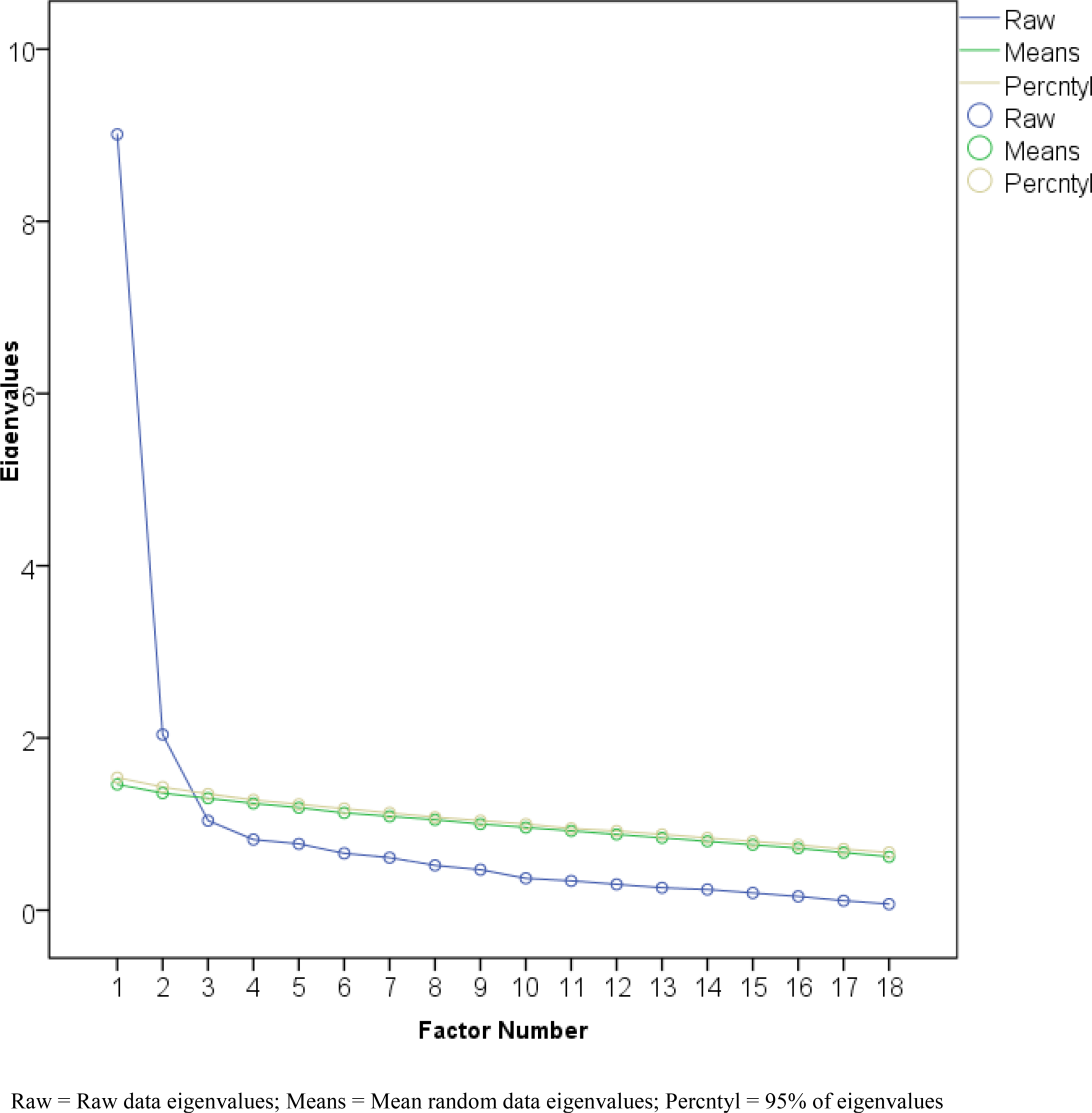
Scree plot illustrating the factor loadings with parallel analysis of the Chinese-Cantonese version of the Fear of Intimacy with helping professionals scale (C-FIS-HP) (N = 297).

The diagram of a scree plot with parallel analysis is shown in Fig 1. The plotting the raw data versus randomly generated eigenvalues provides a visual comparison of the results. Fig 1 shows a plot of the eigenvalues along with the mean and 95th percentiles of the eigenvalues. The C-FIS-HP indicates retaining the two factors whose raw eigenvalues lie above the lines representing the randomly generated eigenvalues [44].

The KMO measure was 0.92, which indicated that the data were applicable for the factor analysis [49]. Bartlett’s test of sphericity supported the factorability of the correlation matrix, with a statistical significance of *P* < 0.001 [48]. According to screen plot with parallel analysis and empirical findings [10, 44, 45], possible number of factor for C-FIS-HP was suggested ranged from 1 to 3. We examined a series of EFA tests including 1-, 2- and 3-factor structures, using PAF extraction procedure as shown in Table 5. We found that the 2-factor model produced the most interpretable construct comparing to 1-factor or 3-factor model for the 297 older people. Among three models, 2-factor model is selected because factor loadings of 18 items are explained by two distinct factors with high communalities [22, 47]. The factor loading of factor one is 0.51 to 0.85 and factor two is 0.40 to 0.66. A two-factor model was determined to explain 56.25% of the total variance, which is considered sufficient for a coherent construct of C–FIS–HP. After analyzing the meanings of items of the two factors, we named these two factors as “willingness to share” and “fear of sharing” to make empirical and theoretical sense as indicators of a coherent construct of C–FIS–HP (see Table 5).

**Table 5:** Comparisons of 1-factor, 2-factor and 3-factor models of the C-FIS-HP using principal axis factoring extraction (n = 297).

		3-factor			2-factor		1-factor
		Factor 1	Factor 2	Factor 3	Factor 1	Factor 2	Factor 1
FIS15	Comfortable telling HP my needs	**0.87**	0.18	-0.05	**0.85**	0.19	**0.86**
FIS16	Comfortable about having open and honest communication	**0.87**	0.20	-0.06	**0.84**	0.22	**0.86**
FIS11	Comfortable trusting HP with deepest thoughts	**0.81**	0.19	0.25	**0.84**	0.19	**0.85**
FIS12	Comfortable revealing my shortcomings	**0.81**	0.21	0.16	**0.83**	0.21	**0.84**
FIS10	Comfortable telling HP things do shared with others	**0.77**	0.23	**0.55**	**0.82**	0.22	**0.85**
FIS9	Comfortable sharing personal information	**0.77**	0.26	**0.42**	**0.81**	0.25	**0.85**
FIS5	Comfortable discussing significant problems with HP	**0.82**	0.16	-0.07	**0.80**	0.18	**0.80**
FIS8	Not afraid to share what I dislike about myself	**0.79**	0.18	0.05	**0.79**	0.18	**0.80**
FIS18	Comfortable talking about personal goals	**0.78**	0.16	0.09	**0.79**	0.17	**0.79**
FIS6	Comfortable telling experiences, even sad ones, to HP	**0.77**	0.18	0.12	**0.78**	0.19	**0.80**
FIS3	Comfortable expressing my true feelings	**0.74**	0.19	-0.06	**0.72**	0.20	**0.74**
FIS17	Would feel at ease to be myself with HP	**0.53**	0.28	-0.08	**0.51**	0.29	**0.58**
FIS13	Afraid of sharing private thoughts	0.19	**0.65**	-0.11	0.17	**0.66**	0.39
FIS4	Afraid to confide innermost feelings	0.15	**0.64**	0.07	0.15	**0.65**	0.38
FIS7	Difficult being open with HP	0.33	**0.61**	0.01	0.32	**0.62**	**0.52**
FIS14	Afraid might not feel close to HP	0.28	**0.62**	-0.20	0.25	**0.61**	**0.45**
FIS1	Uncomfortable telling about shameful things	0.06	**0.47**	0.15	0.08	**0.46**	0.24
FIS2	Uneasy talking with HP about something that has hurt me deeply	0.02	**0.42**	0.23	0.05	**0.40**	0.20
Eigenvalues		8.71	1.49	0.606	8.66	1.46	-
Percent variance explained		48.36%	8.27%	3.37%	48.12%	8.13%	-
Total variance explained		60.00%			56.25%		47.92%

Kaiser-Olkin Measure of Sampling Adequacy = 0.92; Bartlett’s Test of Sphericity < .0001; HP = the person who would be in the professional helping with you

C-FIS-HP = Chinese version of the Fear of Intimacy with Helping Professionals scale; Bold means factor loading >0.4.

### Confirmatory factor analysis

**Fig 2:**
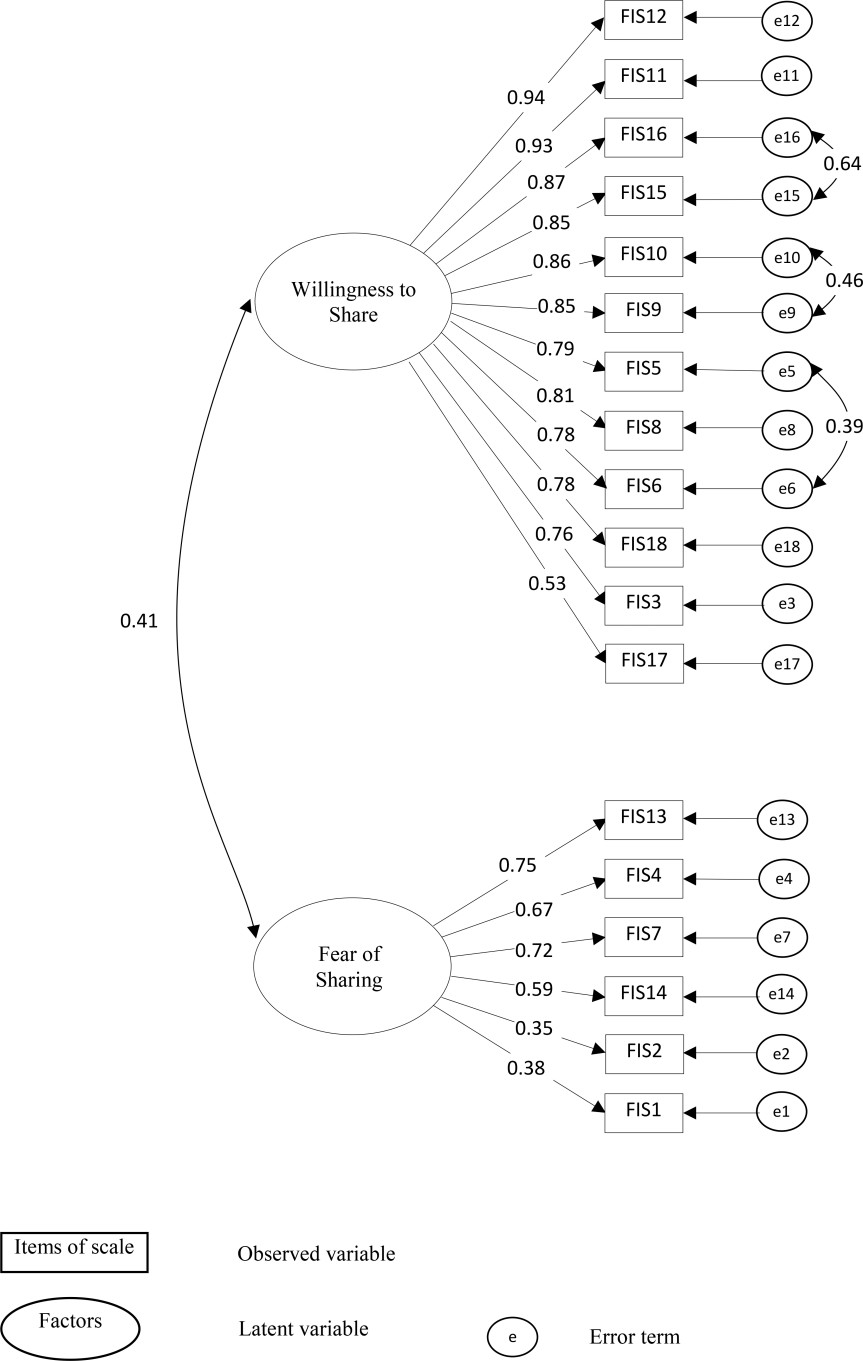
Two-factor model of Chinese-Cantonese version of the Fear of Intimacy with helping professionals scale (C-FIS-HP) (N = 296).

**Fig 3:**
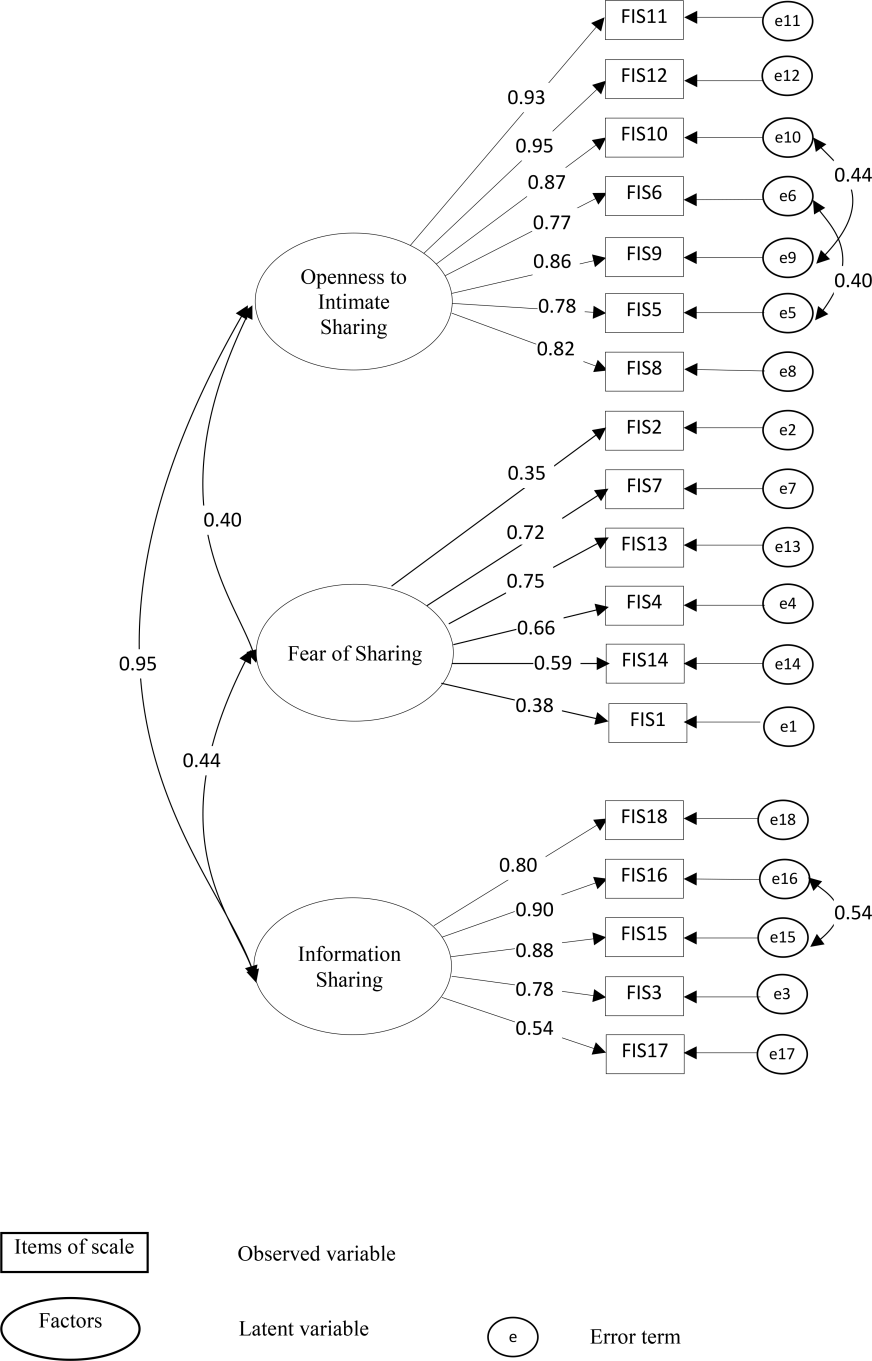
Three-factor model of Chinese-Cantonese version of the Fear of Intimacy with helping professionals scale (C-FIS-HP) (N = 296).

A first-order CFA was used to confirm the construct dimensionality of C–FIS–HP whether the data were consistent with the 2-factor model that had been suggested by the EFA among a second set of older people (n = 296) using AMOS 24.0 software [48]. Fig 2 shows a 2-factor model of C-FIS-HP. Given 3-factor model of C–FIS–HP in previous suggested construct dimensionality [10], we conduct a CFA for 3-factor model of C–FIS–HP using same items in three factors of previous study [10] among our sample (n = 296) as shown in Fig 3. To further understand the structure of C-FIS-HP, we used a bi-factor model with one general factor and three specific factors as well as one general factor and two special factors of C–FIS–HP. Results showed that a bi-factor structure was not a good fit to helping relationship from current sample.

Table 6 compares model fit statistics between 2-factor and 3-factor models of C-FIS-HP. The initial model suggested a fair fit; hence, we try to improve model fit by modification indices [50]. An inspection of the modification indices suggested that the correlations among three-error terms 5 and 6, 9 and 10, and 15 and 16 are additional free parameters that could improve the model. We investigated the meaning of these items. We compared the item 5 (Comfortable discussing significant problem with HP) and item 6 (Comfortable telling experiences, even sad one, to HP); item 9 (Comfortable sharing personal information) and item 10 (comfortable telling HP not shared with others); item 15 (Comfortable telling HP my needs) and item 16 (Comfortable having open and honest communication with HP). The inter-item correlations between item 5 and 6; item 9 and 10; and item 15 and 16 were 0.77; 0.88 and 0.89, indicating that items are measuring the same underlying characteristic. We found the meanings for three pairs were similar interpretation in Chinese language so we allowed a covariance between two error items [50]. After the modification, the modified model and the CFA demonstrated a satisfactory fit to the data [53, 54] for both 2-factor and 3-factor models (see Figs 2 and 3). Factor loadings in the two models were all significant (*P* < 0.01). However, we found the correlations estimate between factor 1 “Openness to Intimate Sharing” and factor 3 “Information Sharing” were very high (correlation coefficient, *r* = 0.95). It is possible that all items may explained by one factor rather than two factors [50]. Therefore, a two-factor model was selected because it had the best empirical and conceptual fit to the data (NFI = 0.92, IFI = 0.95, TLI = 0.94, CFI = 0.95, and RMSEA = 0.07) as shown in Fig 3. The factor loading of “willingness to share” ranged from 0.53 to 0.94 and “fear of sharing” ranged from 0.38 to 0.75.

**Table 6:** Comparisons of model fit statistics between 2-factor and 3-factor models (n = 296).

	Model goodness-of fit indices						
Model						90% RMSEA	
	NFI	IFI	TLI	CFI	RMSEA	Low	High
2-factor							
Initial model	0.86	0.89	0.88	0.89	0.11	0.10	0.12
Modified model	0.92	0.95	0.94	0.95	0.07	0.06	0.08
3-factor							
Initial model	0.89	0.93	0.91	0.93	0.09	0.08	0.10
Modified model	0.93	0.96	0.95	0.96	0.07	0.06	0.08

## Discussion

The current study provides empirical support to the reliability and validity of C–FIS–HP as a tool for measuring helping relationships of older Chinese people in Macau. In terms of reliability testing, the internal consistency of C–FIS–HP was supported by satisfactory findings from Spearman-Brown split half reliability and Cronbach’s alpha; this result was consistent with that of a previous study [10]. Our study demonstrated the high item-to-total correlation between the items and their subscales to support the homogeneity of C–FIS–HP. In addition, the high ICC that was determined over a three-week period supports the high stability of C–FIS–HP over time. In summary, the reliability of C–FIS–HP in our study was good.

Consistent with other studies [7,8], the convergent validity of C–FIS–HP was confirmed by the negative relationship between the scores of C–ESCAS and the C–FIS–HP. Therefore, those who had a considerably high self-care ability showed significantly high comfort level in disclosing intimate details about their life to a helping professional [7,8]. The different levels of medication adherence and multiple chronic condition groups in C–FIS–HP were discriminated through known-group comparisons analysis. The results revealed significant differences among the subjects of the three levels of medication adherence and between the subjects of the two groups with chronic conditions. These findings indicated that older people who had considerably high comfort level in disclosing intimate details about their life to a helping professional were also likely to be in the high medication-adherence group [9] and multiple chronic conditions [40,41].

In the current study, the structural validity showed a two-factor structure by EFA and CFA in two separate samples. These results were inconsistent with a three-factor structure by EFA in a previous study, namely, “fear of sharing,” “openness to intimate sharing,” and “information sharing,” in Mainland Chinese populations [10]. The strongest factor in the present study was “willingness of sharing,” whereas in another study, the strongest factors were the combined items of two factors, namely, “openness to intimate sharing” and “information sharing” [10]. However, the items for the second factor, which was “fear of sharing” in this study, had identical items in the previous study [10]. The difference in the factor structure may associate with the different help-seeking attitudes toward professionals across cultures [11,12] between Mainland China [10] and our study in Macau. The discrepancy may also be due to the differences in age group, gender proportion, and educational level between our study compared with the previous one [10], thereby possibly influencing the interpretation of the items of the scale. Indeed, the two factors reflected the sense of sharing with helping professionals in the positive and negative dimensions [2]. These two factors highlight cultural differences in professional help seeking and awareness of these cultural difference are crucial for successful clinical service [11]. The finding of this study may help to design our service in a cultural-specific way. Hence, use of culturally normative communication is vital in improving treatment outcomes [11]. These two dimensions may provide meaningful clinical parameters that may be beneficial for a considerably accurate assessment of the helping relationship. Given that a different factorial structure emerged from this study, further testing in different Chinese populations using a multidimensional approach is necessary to confirm the structure.

## Implications

The helping relationship is essential for improving health outcomes [5,17]; thus, a scientifically sound clinical tool is beneficial for assessing helping relationship. Our study shows that C–FIS–HP has satisfactory reliability and validity that may have clinical and educational implications. Health professionals can use C–FIS–HP as a clinical tool to assess the comfort level of patients in a helping relationship, as well as use this information to develop culturally sensitive therapeutic interventions and treatment plans. Moreover, understanding the helping relationship can be an excellent method for health professionals to reflect on their communication skills [55] and develop in-service training and educational programs to enhance therapeutic relationships and the quality of their services [6]. We believe that further use of this instrument will allow additional sophisticated data analysis techniques for further validation and generalizability of C–FIS–HP in a variety of settings. C–FIS–HP can also provide opportunities for future cross-cultural comparative studies. Such studies will enable the evaluation of the universality of constructs of C–FIS–HP.

## Limitations

Caution is necessary in interpreting the findings of this study in light of a few limitations. First, one limitation was linked to the administration of the questionnaire through face-to-face interview, which may have delivered biased and socially desirable responses. Second, we adopted a cross-sectional design solely in community settings, thereby possibly limiting the generalizability of the results. A long-term longitudinal cohort study may be considered to measure the predictive validity of C–FIS–HP. Third, we noticed the inter-item correlations of between item 5 and 6; item 9 and 10; and item 15 and 16 were above 0.50 so the construct of three pairs tend to be very similar to each other. Further studies are warrant to examine these items to prevent redundancy. Further psychometric tests, including concurrent, divergent validity, and equivalent (parallel or alternate) form are encouraged in different age groups.

## Conclusions

Overall, the results of this study verified that C–FIS–HP is a psychometrically sound measurement tool. Further studies that concern other forms of reliability and validity, as well as the application of C–FIS–HP in various regions, need to be conducted to facilitate the eventual application of this tool to Chinese nationals around the world.

## Supporting information

S1 File Data set for PLOS one (n = 593). (SAV)

## References

[1] Capuzzi D, Stauffer MD, Gross DR (2016) The helping relationship: From core dimensions to brief and integrative possibilities Counseling and Psychotherapy: Theories and Interventions (6th edition). Alexandria: Wiley Publishers.

[2] Boyer CA, Lutfey KE (2010) Examining critical health policy issues within and beyond the clinical encounter: Patient-provider relationships and help-seeking behaviors. J Health Soc Behav 51: S80–S93. doi: 10.1177/0022146510383489 20943585 10.1177/0022146510383489

[3] Riley JB (2017) Communication in nursing (8th Edition). St. Louis: Elsevier.

[4] DiMatteo MR, Haskard-Zolnierek KB, Martin LR (2012) Improving patient adherence: a three-model to guide practice. Health Psychol Rev 6: 74–91. doi: 10.1080/17437199.2010.537592

[5] Kelley JM, Kraft-Todd G, Schapira L, Kossowsky J, Riess H (2014) The influence of the patient-clinician relationship on healthcare outcomes: a systematic review and meta-analysis of randomized controlled trials. PLoS One 9: e94207 doi: 10.1371/journal.pone.0094207 24718585 10.1371/journal.pone.0094207 PMC3981763

[6] Petrakis M, Brophy L, Lewis J, Stylanou M, Scott M, Cocks N, et al (2014) Consumer measures and research co-production: A pilot study evaluating the recovery orientation of a mental health program collaboration. Asia Pac J Soc Work 24: 94–108. doi: 10.1080/02185385.2014.885212

[7] DeVito Dabbs A, Terhorst L, Song MK, Shellmer DA, Aubrecht J, Connolly M, et al (2013) Quality of recipient-caregiver relationship and psychological distress are correlates of self-care agency after lung transplantation. Clin Transplant 27: 113–120. doi: 10.1111/ctr.12017 23004565 10.1111/ctr.12017 PMC3530624

[8] Shrivastava SR, Shrivastava PS, Ramasamy J (2013) Role of self-care in management of diabetes mellitus. J Diabetes Metab Disord 12:14 doi: 10.1186/2251-6581-12-14 23497559 10.1186/2251-6581-12-14 PMC3599009

[9] Bauer AM, Parker MM, Schiliger D, Katon WJ, Adler N, Adams AS, et al (2014) Associations between antidepressant adherence and shared decision-making, patient-provider trust, and communication among adults with diabetes: diabetes study of Northern California (DISTANCE). J Gen Intern Med 29:1139–1147. doi: 10.1007/s11606-014-2845-6 24706097 10.1007/s11606-014-2845-6 PMC4099457

[10] Ingersoll TS, Poulin J, Deng R, Shan X, Witt H, Swain M, et al (2012) Fear of intimacy with helping professionals scale: reliability and validity of English and Mandarin versions. J Evid Based Soc Work 9: 317–332. doi: 10.1080/15433714.2010.516689 22830935 10.1080/15433714.2010.516689

[11] Mojaverian T, Hashimoto T, Kim HS (2013) Cultural differences in professional help seeking: a comparison of Japan and the U.S. Front Psychol 3: 615 doi: 10.3389/fpsyg.2012.00615 23426857 10.3389/fpsyg.2012.00615 PMC3576055

[12] Matsumoto D, Juang L (2017) Culture and psychology (6th Edition). Canada: Nelson Education.

[13] Fang T, Faure GO (2011) Chinese communication characteristics: A Ying Yang perspective. Int J Intercult Rel 35: 320–333. doi: 10.1016/j.ijintrel.2010.06.005

[14] Hsu CHC, Huang S (2016) Reconfiguring Chinese cultural values and their tourism implications. Tourism Manage 54: 230–242. doi: 10.1016/j.tourman.2015.11.011

[15] Fang K, Pieterse AL, Friedlander M, Cao J (2011) Assessing the psychometric properties of the attitudes toward seeking professional psychological help scale-short form in mainland China. Int J Adv Counselling 33: 309–321. doi: 10.1007/s10447-011-9137-1

[16] Qi GY, Zeng SX, Shi JJ, Meng XH, Lin H, Yang QX, et al (2014) Revisiting the relationship between environmental and financial performance in Chinese industry. J Environ Manage 145: 349–356. doi: 10.1016/j.jenvman.2014.07.010 25113229 10.1016/j.jenvman.2014.07.010

[17] Witt H, Poulin J, Ingersoll T, Deng R (2011) Older Chinese adults' fear of intimacy with helping professionals. J Cross Cult Gerontol 26: 71–83. doi: 10.1007/s10823-010-9132-8 21161356 10.1007/s10823-010-9132-8

[18] Chen HF, Tsai YF, Lin YP, Shih MS, Chen JC (2010) The relationships among medicine symptom distress, self-efficacy, patient-provider relationship, and medication compliance in patients with epilepsy. Epilepsy Behav 19: 43–49. doi: 10.1016/j.yebeh.2010.06.007 20719572 10.1016/j.yebeh.2010.06.007

[19] Khanfar NM, Zapantis A, Alkhateeb FM, Clauson KA, Beckey C (2013) Patient attitudes toward community pharmacist attire. J Pharm Pract 26: 442–447. doi: 10.1177/0897190012465956 23184409 10.1177/0897190012465956

[20] Bodenlos JS, Grothe KB, Whitehead D, Konkle-Parker DJ, Jones GN, et al (2007) Attitudes toward health care providers and appointment attendance in HIV/AIDS patients. J Assoc Nurses AIDS Care 18: 65–73. doi: 10.1016/j.jana.2007.03.002 17570301 10.1016/j.jana.2007.03.002

[21] Descutner CJ, Thelen MH (1991) Development and validation of a fear-of-intimacy scale. Psychol Assessment 3: 218–225. org/10.1037/1040-3590.3.2.218

[22] Fabrigar LR, Wegener DT, MacCallum RC, Strahan EJ (1999) Evaluating the use of exploratory factor analysis in psychological research. Psychol Methods 4: 272–299.

[23] Harrington D (2009) Confirmatory factor analysis New York: Oxford University Press.

[24] Groth-Marnat G, Wright AJ (2016) Handbook of Psychological Assessment (6th Edition). New York: Wiley.

[25] Macao Statistics and Census Service (2015) Yearbook of statistics Macao: Statistics and census service Macao SAR government.

[26] Reynolds K, Pietrzak RH, El-Gabalawy R, Mackenzie CS, Sareen J (2015) Prevalence of psychiatric disorders in U.S. older adults: findings from a nationally representative survey. World Psychiatry 14: 74–81. doi: 10.1002/wps.20193 25655161 10.1002/wps.20193 PMC4329900

[27] Freedman VA, Grafova IB, Rogowski J (2011) Neighborhoods and chronic disease onset in later life. Am J Public Health 101: 79–86. doi: 10.2105/AJPH.2009.178640 20299643 10.2105/AJPH.2009.178640 PMC2912970

[28] MacCallum RC, Widaman KF, Preacher KJ, Hong S (2001) Sample Size in factor analysis: The role of model error. Multivariate Behav Res 36: 611–637. doi: 10.1207/S15327906MBR3604_06 26822184 10.1207/S15327906MBR3604_06

[29] Shah R, Goldstein SM (2006) Use of structural equation modeling in operations management research: Looking back and forward. J Oper Manag 24: 148–169. doi: 10.1016/j.jom.2005.05.001

[30] Kearney BY, Fleischer BJ (1979) Development of an instrument to measure exercise of self-care agency. Res Nurs Health 2: 25–34. doi: 10.1002/nur.4770020105 254279 10.1002/nur.4770020105

[31] Wang HH, Laffrey SC (2000) Preliminary development and testing of instruments to measure self-care agency and social support of women in Taiwan. Kaohsiung J Med Sci 16: 459–467. 11271731

[32] Wong CL, Ip WY, Shiu TY (2012) Translation and validation of the Chinese-Cantonese version of the exercise of self-care agency scale. Int J Nurs Stud 49: 1122–1137. doi: 10.1016/j.ijnurstu.2012.04.004 22572019 10.1016/j.ijnurstu.2012.04.004

[33] Morisky DE, Green LW, Levine DM (1986) Concurrent and predictive validity of a self-reported measure of medication adherence. Med Care 24: 67–74. 3945130 10.1097/00005650-198601000-00007

[34] Tzeng JI, Chang CC, Chang HJ, Lin CC (2008) Assessing analgesic regimen adherence with the Morisky Medication Adherence Measure for Taiwanese patients with cancer pain. J Pain Symptom Manage 36: 157–166. doi: 10.1016/j.jpainsymman.2007.10.015 18411015 10.1016/j.jpainsymman.2007.10.015

[35] Terwee CB, Bot SD, de Boer MR, van der Windt DA, Knol DL, Dekker J, et al (2007) Quality criteria were proposed for measurement properties of health status questionnaires. J Clin Epidemiol 60: 34–42. doi: 10.1016/j.jclinepi.2006.03.012 17161752 10.1016/j.jclinepi.2006.03.012

[36] Kline P (2000) The handbook of psychological testing New York: Routledge.

[37] Houser J (2011) Nursing research: reading, using and creating evidence (2nd edition). Canada: Jones & Bartlett.

[38] Godwin M, Pike A, Bethune C, Kirby A, Pike A (2013) Concurrent and convergent validity of the simple lifestyle indicator questionnaire. ISRN Family Med 2013: 529645 doi: 10.5402/2013/529645 24967324 10.5402/2013/529645 PMC4041224

[39] Potrney LG, Watkins MP (2000) Foundations of clinical research: Applications to practice Upper Saddle River, NJ: Prentice Hall Health.

[40] Jabaaij L, Akker M, Schellevis F (2012) Excess of health care use in general practice and of comorbid chronic conditions in cancer patients compared to controls. BMC Fam Pract 13: 1–7. 22712888 10.1186/1471-2296-13-60 PMC3480891

[41] Bähler C, Huber CA, Brüngger B, Reich O (2015) Multimorbidity, health care utilization and costs in an elderly community-dwelling population: a claims data based observational study. BMC Health Serv 15: 23 doi: 10.1186/s12913-015-0698-2 25609174 10.1186/s12913-015-0698-2 PMC4307623

[42] Fuertes JN, Mislowack A, Bennett J, Paul L, Gilbert TC, Fontan G, et al (2007) The physician–patient working alliance. Patient Educ Couns 66: 29–36. doi: 10.1016/j.pec.2006.09.013 17188453 10.1016/j.pec.2006.09.013

[43] Kane JM, Kishimoto T, Correll CU (2013) Non-adherence to medication in patients with psychotic disorders: epidemiology, contributing factors and management strategies. World Psychiatry 12: 216–226. doi: 10.1002/wps.20060 24096780 10.1002/wps.20060 PMC3799245

[44] O'Connor BP (2000) SPSS and SAS programs for determining the number of components using parallel analysis and Velicer's MAP test. Behav Res Methods Comput 32 (3): 396–402. doi: 10.3758/BF03200807 10.3758/bf03200807 11029811

[45] Cattell RB (1966) The scree test for number of factors. Multivar Behav Rese 1(2): 245–276. doi.org/10.1207/s15327906mbr0102_10 10.1207/s15327906mbr0102_10 26828106

[46] Nunnally JC, Bernstein IH (1994) Psychometric theory New York: McGraw-Hill.

[47] Streiner DL, Norman GR (2008) Health measurement scales: a practical guide to their development and use Oxford, New York: Oxford University Press.

[48] Bartlett MS (1954) A note on the multiplying factors for various Chi-square approximations. J R Stat Soc 16: 296–298.

[49] Hair J, Black WC, Babin BJ, Anderson RE (2010) Multivariate data analysis (7th edition). Englewood Cliffs, NJ: Prentice Hall.

[50] Byrne BM (2016) Structural equation modeling with AMOS: Basic concepts, applications, and programming (3rd edition). New York, NY: Routledge.

[51] Chen F. F., Hayes A., Carver C. S., Laurenceau J. P., Zhang Z. (2012). Modeling general and specific variance in multifaceted constructs: A comparison of the bifactor model to other approaches. J Pers 80: 219–251. doi: 10.1111/j.1467-6494.2011.00739.x 22092195 10.1111/j.1467-6494.2011.00739.x

[52] Tabachnick BG (2007) Using multivariate statistics (5th edition). Boston: Pearson.

[53] Browne MW, Cudeck R (1992) Alternative ways of assessing model fit. Socio Meth Res 21: 230–258. doi: 10.1177/0049124192021002005

[54] Schermelleh-Engel K, Moosbrugger H (2003) Evaluating the fit of structural equation models: Test of significance and descriptive goodness-of-fit measures. Meth Psychol Res Online 8: 23–74.

[55] Fuertes JN, Toporovsky A, Reyes M, Osborne JB (2016) The physician-patient working alliance: Theory, research, and future possibilities. Patient Educ Couns (In Press) doi: 10.1016/j.pec.2016.10.018 27773600 10.1016/j.pec.2016.10.018

