# Correction: Psychometric evaluation of the Chinese version of the fear of intimacy with helping professionals scale

**DOI:** 10.1371/journal.pone.0204440

**Published:** 2018-09-17

**Authors:** The PLOS ONE Staff

The affiliation and current address for the first author are swapped. Affiliation 1 should be: School of Health Sciences, Macao Polytechnic Institute, Macau Special Administration Region, China. The current address of Ying Lau should be: Department of Alice Lee Centre for Nursing Studies, Yong Loo Lin School of Medicine, National University of Singapore, Singapore. The publisher apologizes for the errors.
